# Expression of CD274 mRNA Measured by qRT-PCR Correlates With PD-L1 Immunohistochemistry in Gastric and Urothelial Carcinoma

**DOI:** 10.3389/fonc.2022.856444

**Published:** 2022-04-27

**Authors:** So Young Kang, You Jeong Heo, Ghee Young Kwon, Kyoung-Mee Kim

**Affiliations:** ^1^Department of Pathology and Translational Genomics, Samsung Medical Center, Sungkyunkwan University School of Medicine, Seoul, South Korea; ^2^The Samsung Advanced Institute for Health Sciences & Technology (SAIHST), Samsung Medical Center, Sungkyunkwan University School of Medicine, Seoul, South Korea; ^3^Center of Companion Diagnostics, Samsung Medical Center, Seoul, South Korea

**Keywords:** mRNA expression, immunotherapy, gastric, urothelial, CD274, carcinoma

## Abstract

Programmed death-ligand 1 (PD-L1) immunohistochemistry (IHC) is widely used to predict the clinical responses to immune checkpoint inhibitors (ICIs). However, PD-L1 IHC suffers from the complexity of multiple testing platforms and different cutoff values caused by the current one drug-one diagnostic test co-development approach for ICIs. We aimed to test whether PD-L1 (CD274) mRNA expression levels measured using quantitative reverse transcription-polymerase chain reaction (qRT-PCR) can represent PD-L1 IHC and predict responses to ICI. The FDA-approved PD-L1 IHC results with 22C3 pharmDx (gastric cancer) and SP142 (urothelial carcinoma) were compared with *CD274* mRNA expression levels via qRT-PCR using the same formalin-fixed, paraffin-embedded tissue blocks from 59 gastric cancer and 41 urothelial carcinoma samples. *CD274* mRNA expression was identified using three independent sets of primers and TaqMan® probes targeting exon 1–2, exon 3–4, and exon 5–6. *CD274* mRNA levels in spanning exon 1–2, exon 3–4, and exon 5–6 junctions of *CD274* correlated well with PD-L1 expression (r^2^=0.81, 0.65, and 0.59, respectively). The area under the curve of exon 1–2 was the highest (0.783), followed by exon 3–4 (0.701), and exon 5–6 (0.671) of the *CD274* gene against the PD-L1 combined positive score cutoff of 10. When *CD274* mRNA expression was matched for response to immunotherapy, the overall response rate was higher in patients with high *CD274* mRNA levels with a cutoff of 0.0722 (gastric cancer) and 0.0480 (urothelial carcinoma) than in those with low *CD274* mRNA expression (*P* < 0.001 and P = 0.018, respectively). These results show that *CD274* mRNA levels predicted ICI responses in patients with gastric or urothelial carcinomas and could be used as alternatives for PD-L1 IHC.

## Introduction

In 2017, the Food and Drug Administration (FDA) granted accelerated approval to pembrolizumab for patients with recurrent locally advanced or metastatic, gastric or gastroesophageal junction adenocarcinoma whose tumors express programmed death-ligand 1 (PD-L1) as determined by an FDA-approved test based on the clinical results of KEYNOTE 059 (NCT02335411) (1). In advanced gastric or gastroesophageal junction adenocarcinoma, PD-L1 expression is assessed using the FDA-approved PD-L1 IHC 22C3 pharmDx assay and a combined positive score (CPS) (2). In 2016, FDA gave accelerated approval to atezolizumab injection (Tecentriq) for the treatment of patients with locally advanced or metastatic urothelial carcinoma who have disease progression during or following platinum-containing chemotherapy or have disease progression within 12 months of neoadjuvant or adjuvant treatment with platinum-containing chemotherapy. FDA approved Ventana PD-L1 (SP142) assay to measure PD-L1 expression in urothelial carcinoma. With FDA approvals, PD-L1 immunohistochemistry (IHC) is popular for predicting therapeutic responses to immune checkpoint blockade (ICB) (3). While this method measures PD-L1 protein levels, antibody clones, staining platforms, and interpretations differ. For instance, whereas that in metastatic non-small cell lung cancer (NSCLC) samples relies on tumor proportion scores (TPS) instead of CPS (4). The Ventana SP142 assay is used to analyze urothelial carcinoma (UC) and to count immune cells (IC) within the tumor microenvironment (5). This variability in scoring methods has contributed to confounding results across clinical trials and in clinical practice, leading to uncertainty regarding the universal value of PD-L1 protein levels as a biomarker across tumor types (6, 7). Furthermore, the use of formalin-fixed, paraffin-embedded (FFPE) archival tumor tissues prepared, fixed, and stored in non-standardized ways might not generate predictable and intended results for adequate PD-L1 antigen retrieval. This could potentially increase the heterogeneity of IHC intensity, extent, and topography of staining (3). All these factors complicate the reliability of PD-L1 levels assessed by IHC to predict clinical responses to ICB (8).

Assays of FFPE tissues based on RNA are currently employed clinically to classify or predict recurrence risk in patients affected by various types of tumors (9, 10). Gene expression assays based on RNA include microarray, real-time quantitative reverse transcription polymerase chain reaction (qRT-PCR), and RNA sequencing (11–13). The qRT-PCR assays are popular for quantifying genes due to a large dynamic range, high sensitivity, high specificity, little to no post-amplification processing, and increased sample throughput (14, 15). The use of specific primers targeting stably expressed genes provides high specificity and sensitivity, allowing for the simultaneous measurement of several targets, including genes, for sample quality control purposes. Gene expression profiling by qRT-PCR has minimal input requirements and could be far more cost-effective than IHC. Furthermore, close concordance between qRT-PCR and IHC has validated qRT-PCR analyses, even for challenging FFPE tumor samples (16). Therefore, gene-specific reverse transcription might considerably increase the success rate of molecular classifier validation in FFPE sample cohorts.

The present study aimed to develop a more rapid qRT-PCR assay to measure *CD274* mRNA expression that closely correlates with PD-L1 IHC and save archival tumor tissues for other IHC assays in the same patient. Therefore, we designed three qRT-PCR primers and compared their results with those of PD-L1 IHC, then clinically validated the results in patients with GC and UC treated with ICIs.

## Materials and Methods

### Patients and Data Collection

We collected retrospective data from 100 patients with advanced GC (n = 59) or UC (n = 41) that were treated with palliative chemotherapy (n = 100) and anti-programmed death 1 (PD-1)/PD-ligand (L)-1 immunotherapy (n = 49) at Samsung Medical Center between December 2016 and January 2020. The median age was 61.0 (33–81) years and 30 (61.2%) patients were male. All the patients present with GC were stage IIB–IV disease at diagnosis and have experienced local recurrence or metastasis at treatment for ICI. For UC patients, they were all locally advanced stage II–IIIb disease stages (**Supplementary Table S1**). Responses of the 49 patients treated with immunotherapy were assessed every 6–12 weeks according to the Immune Response Evaluation Criteria in Solid Tumors (iRECIST) (17). Data from patients with at least 6 weeks of follow up were included. The primary clinical endpoint was the objective response rate (ORR), defined as a complete (CR) or partial (PR) response. Patients with progressive (PD) or stable (SD) disease were classified as non-responders. Clinicopathological data were retrospectively extracted from electronic medical records. This study proceeded in accordance with the Institutional Review Board guidelines (IRB No. 2018-09-041-001) for data analysis and investigational treatment, and written informed consent from the patients was also obtained to analyze their innominate data.

### RNA Extraction and qRT-PCR

Total RNA was isolated from FFPE tumor tissues using the ReliaPrep™ FFPE Total RNA Miniprep System (Promega Corp., Madison, WI, USA), and a amplified using a high-capacity cDNA reverse transcription kit (Thermo Fisher Scientific Inc., Waltham, MA, USA) as described by the manufacturer. Target genes were analyzed using a gene expression assay with forward and reverse primers and an Applied Biosystems FAM-labeled MGB TaqMan^TM^ probe (Thermo Fisher Scientific Inc.) as we previously described (18). We found that the PD-L1 IHC results correlated with those of NanoString nCounter assays (19), we used *CD274* TaqMan probes spanning exon 1–2 (assay ID; Hs01125296_m1), 3–4 (assay ID; Hs00204257_m1), and 5–6 (assay ID; Hs01125301_m1) boundaries for qRT-PCR (**Supplementary Figure S1**). These sequences were amplified by PCR in triplicate under the following conditions using QuantStudio 6 (Thermo Fisher Scientific Inc.): 2 min at 50°C and 10 min at 94°C, followed by 40 cycles of 95°C for 15 s and 60°C for 60 s. Threshold cycle (Ct) values for each sequence were calculated for each and averaged, and normalized to the mean of the reference gene *GUSB2* (assay ID: Hs99999908_m1), which was stably expressed (18). The mRNA expression of each gene was measured using the 2^^-ΔCt^ (ΔCt = ΔCt_target gene−_ΔCt_GUSB2_) method.

### Immunohistochemical Detection of PD-L1 

Gastric FFPE tissue blocks were cut into 4-μm sections and stained using an Autostainer Link 48 system and Dako PD-L1 IHC 22C3 pharmDx kits (both from Agilent Technologies Inc., Santa Clara, CA, USA) (2). A rabbit anti-human PD-L1 monoclonal antibody (clone SP142; Ventana Medical Systems, Tucson, AZ, USA) was used as described for UC samples (20). The CPS of PD-L1 expression was calculated as the number of PD-L1-stained GC tumors and ICs divided by the total number of viable tumor cells, multiplied by 100. The concordance rate between qRT-PCR and IHC was evaluated using CPS cut-offs of 1 and 10 for GC. Infiltrative ICs covering ≥ 5 of a UC tumor area were defined as PD-L1-positive. For positive control, we used positive cell lines provided by PD-L1 IHC 22C3 pharmDx and tonsil tissues. For negative control, we used MCF-7 cell lines provided by PD-L1 IHC 22C3 pharmDx. Benign human tonsil is tissue control as it contains both positive and negative staining epithelial and immune cells and can serve as both a positive and negative tissue control for VENTANA PD-L1 (SP142) Assay staining (21).

### Statistical Analyses

We used CPS ≥1 and ≥ 10 for GC, and IC ≥5 for UC to compare IHC with qRT-PCR. To calculate the sensitivity, specificity, positive (PPV) and negative (NPV) predictive values, and accuracy, a positive IHC result was considered as CPS ≥1 or ≥ 10 for GC, and IC ≥ 5 for urothelial carcinoma. Predicted responses based on tumor type, IHC results, and qRT-PCR results were evaluated using logistic regression.

The ORR (CR/PR) and disease control rate (DCR; CR/PR/SD) were compared with the *CD274* mRNA qRT-PCR results using two-tailed unpaired Student t-tests. The diagnostic values of panels were assessed by calculating the area under the receiver operating characteristics (ROC) curve (AUC). Kaplan–Meier estimates of progression-free (PFS) and disease-specific survival (DSS) were compared using log-rank tests. All graphs were generated using GraphPad Prism v. 9.0 (GraphPad Software Inc., San Diego, CA, USA). Statistical significance was set at P < 0.05. All data were statistically analyzed using SPSS software version 27.0 (IBM Corp., Armonk, NY, USA).

## Results

### Comparison of IHC and qRT-PCR Results

**Figure 1:**
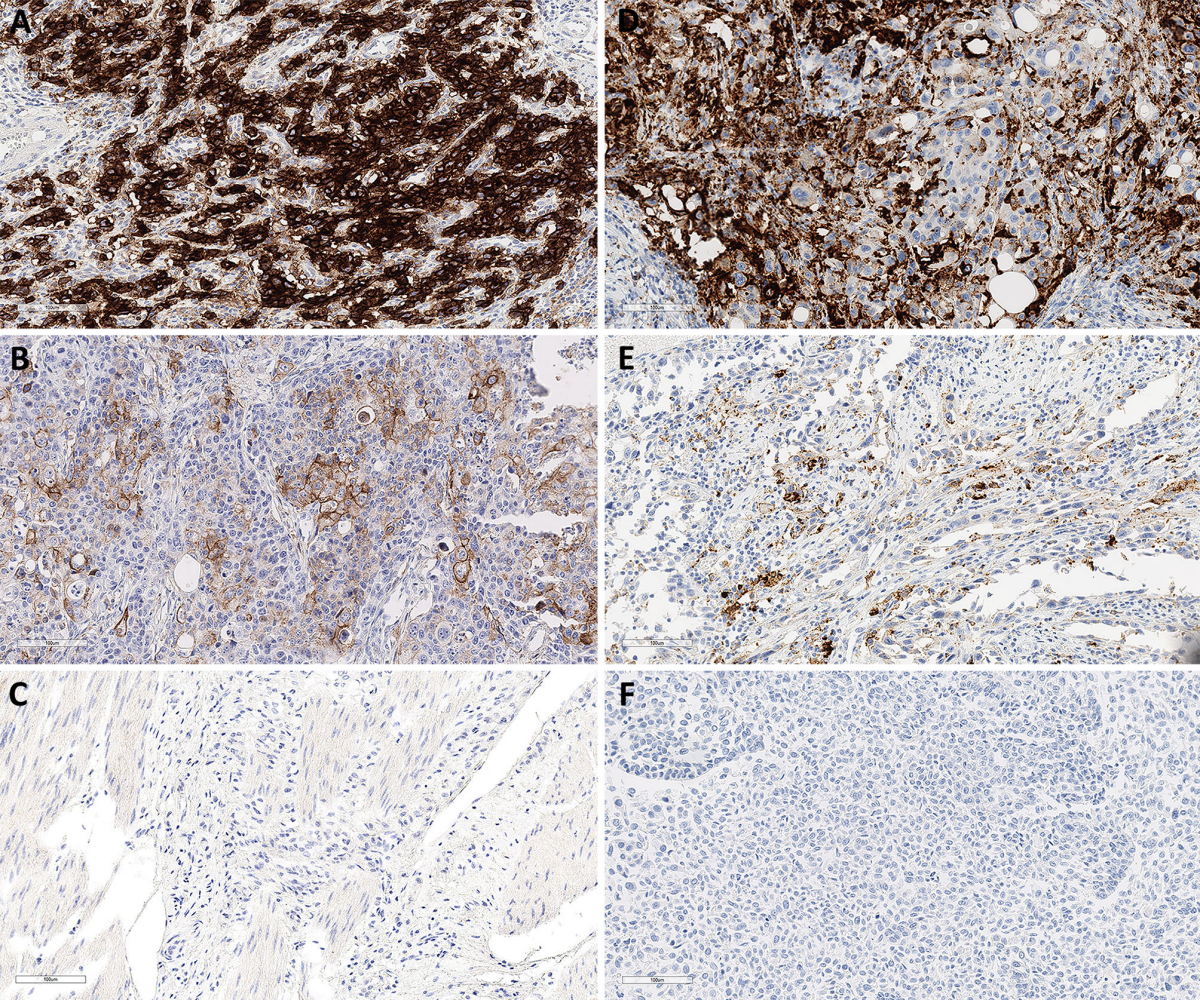
Representative PD-L1 immunohistochemical staining in GC and SP142 in UC. Combined positive scores of 95 **(A)**, 25 **(B)** and 0 **(C)** in GCs with 22C3 pharmDx. Immune cell scores of 40 **(D)**, 20 **(E)** and 0 **(F)** in UCs with Ventana PD-L1 (SP142) assay. Magnification in all images, 20×. GC, gastric cancer; UC, urothelial carcinoma.

The 22C3 pharmDx assay identified PD-L1 positivity with CPS ≥ 1 and ≥ 10 in 32 (54.2%) and 13 (22 %) of 59 GC samples, respectively. The mean PD-L1 CPS in GC was 9.24 (0–95). The Ventana SP142 assay identified PD-L1 positivity with IC ≥ 5 in 12 (29.3%) of 41 UCs. The mean PD-L1 IC in urothelial carcinomas was 10.46 (0–95) (**Figure 1**).

**Figure 2:**
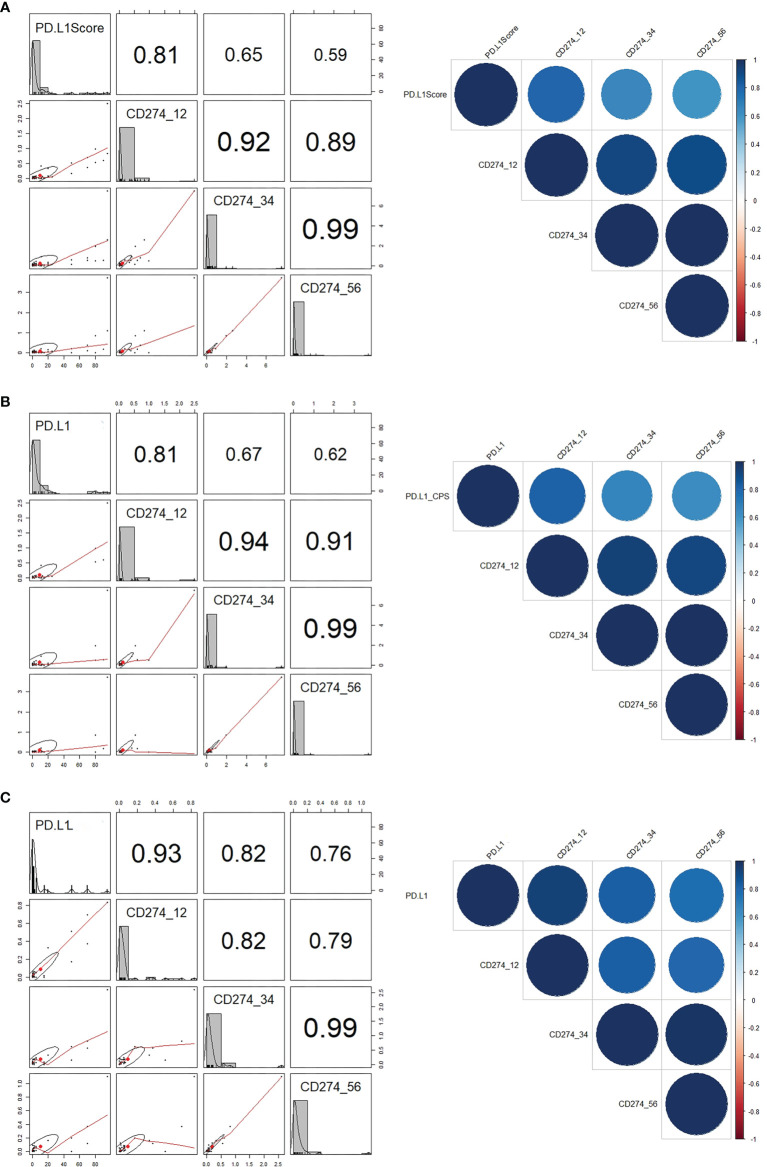
Correlations between PD-L1 scores and *CD274* mRNA expression. **(A)** Correlations between (A) PD-L1 scores and *CD274* exons 1–2, 3–4, and 5–6 in all GC and UC. **(B)** PD-L1 combined positive score and *CD274* mRNA expression in GC. **(C)** PD-L1 immune scores and *CD274* mRNA expression in UC. GC, gastric cancer; PD-L1, programmed death-ligand 1; UC, urothelial carcinoma.

The mean RQ (range) of relative *CD274* mRNA expression spanning exons 1–2, 3–4, and 5–6 were 0.1004 (0–2.4897), 0.2371 (0–7.5214), and 0.0928 (0–3.7064), respectively. These values closely correlated (Spearman correlations: r^2^ = 0.92 for exons 1–2 and 3–4; r^2^ = 0.89 for exons 1–2 and 5–6, and r^2^ = 0.99 for exons 3–4 and 5–6; **Figure 2A**). The PD-L1 scores in 100 evaluated samples closely correlated with *CD274* mRNA expression spanning exons 1–2 (r^2^ = 0.81), 3–4 (r^2^=0.65), and 5–6 (r^2^ = 0.59; **Figure 2A**). In GC, The PD-L1 CPS score with 22C3 pharmDx significantly correlated with the exon 1–2 (r^2^ = 0.81), 3–4 (r^2^ = 0.67), and 5–6 (r^2^ = 0.62) junctions of *CD274* (**Figure 2B**). The Ventana SP142 PD-L1 IC score was significantly associated in UC with exon 1–2 (r^2^ = 0.93), exon 3–4 (r^2^ = 0.82), and exon 5–6 (r^2^ = 0.76) junctions of *CD274* (**Figure 2C**).

The RQ cutoffs of *CD274* mRNA expression in exon 1–2, 3–4, and 5–6 junctions were evaluated as the AUC based on PD-L1 CPS cut-offs of 1 and 10 for GC and PD-L1 IC cut-offs of 5 for UC (**Supplementary Table S2** and **Supplementary Figure S2**). At a CPS cutoff of 10, the highest AUC in GC was 0.783, obtained from *CD274* mRNA expression at the exon 1–2 junction with a cut-off of 0.0722 (P < 0.0001). The highest AUC of UC based on PD-L1 IC cut-offs of IC 5 was 0.781, obtained from *CD274* mRNA expression in the exon 1–2 junction with a cut-off of 0.0480 (P < 0.0001).

### IHC and qRT-PCR Results Predicted Responses to Anti-PD-1/PD-L1 Inhibitor 

Between May 2018 and October 2020, 49 patients were treated with anti PD-1/PD-L1 agents, and treatment responses to treatment with pembrolizumab (n = 16), nivolumab (n = 16), atezolizumab (n = 13), and durvalumab (n = 4) were evaluated during > 6 weeks of followup (**Supplementary Table S1**). The median number PD-1/PD-L1 cycles was 8.9 (range, 1–37) as of May 20, 2021, and the patients were followed up for a median of 11.3 months. **Table 1** summarizes the clinicopathological characteristics of the patients treated with anti-PD-1/PD-L1.

**Table 1:** Clinicopathological characteristics of patients treated with anti-programmed death 1 (PD-1)/programmed death-ligand 1 (PD-L1) therapy.

	Anti-PD-1/PD-L1 patients, No (%)	Overall response rate (CR/PR), (%)	P-value	Disease control rate (CR/PR/SD), (%)	P-value
***Overall***	**49**	**16 (32.7%)**		**30 (61.2%)**	
**Age**			0.261		0.043
<65	30 (61.2%)	8 (26.7%)		15 (50%)	
≥ 65	19 (38.8%)	8 (42.1%)		15 (78.9%)	
**Sex**			0.045		0.001
Male	30 (61.2%)	13 (43.3%)		24 (80%)	
Female	19 (38.8%)	3 (15.8%)		6 (31.6%)	
**Treatment line of** **immunotherapy**			0.929		0.003
1	20 (40.8%)	7 (35%)		18 (90%)	
2	12 (24.5%)	4 (33.3%)		5 (41.7%)	
≥3	17 (34.7%)	5 (29.4%)		7 (41.2%)	
**Immunotherapy** **regimen**			0.196		0.002
Pembrolizumab containing	16 (32.7%)	8 (50%)		12 (75%)	
Nivolumab containing	16 (32.7%)	5 (31.3%)		5 (31.3%)	
Atezolizumab containing	13 (26.5%)	3 (23.1%)		12 (92.3%)	
Durvalumab containing	4 (8.1%)	0 (0%)		1 (25%)	
**Gastric cancer**	**33**	10 (30.3%)		15 (45.5%)	
PD-L1 CPS cutoff 1	18	9 (50%)	0.007	12 (66.7%)	0.007
qRT-PCR cutoff 0.0276	15	6 (40%)	0.269	10 (66.7%)	0.025
PD-L1 CPS cutoff 10	8	5 (62.5%)	0.023	5 (62.5%)	0.266
qRT-PCR cutoff 0.0722	5	5 (100%)	<0.001	5 (100%)	0.008
**Urothelial carcinoma**	**16**	6 (37.5%)		15 (93.8%)	
PD-L1 IC cutoff 5	6	3 (50%)	0.424	5 (83.3%)	0.182
qRT-PCR cutoff 0.0480	5	4 (80%)	0.018	5 (100%)	0.486

qRT-PCR, quantitative reverse transcription-polymerase chain reaction; CR, complete response; PR, partial response; SD, stable disease; PD, progressive disease; CPS, combined positive score; Bold, a statistically significant correlation with a p-value less than 0.05.

**Figure 3:**
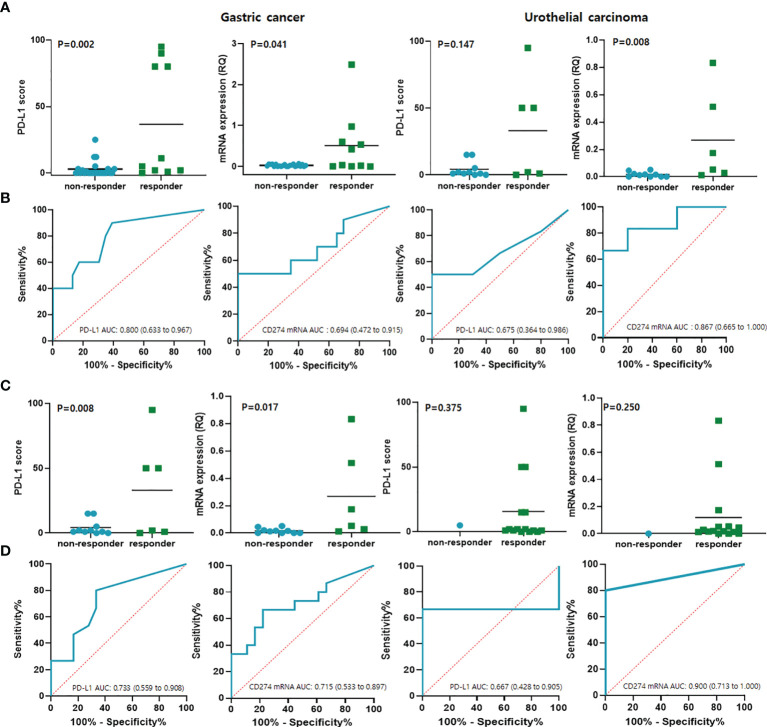
Results of qRT-PCR predicted responses to anti- PD-1 checkpoint blockade in GC and UC. **(A)** PD-L1 and CD274 mRNA expression per iRECIST ORR categories of responders (CR/PR) and non-responders (PD/SD). **(B)** Predictive performance of PD-L1 and CD274 mRNA expression determined from ROC curves in terms of ORR categories. **(C)** PD-L1 and CD274 mRNA expression levels per iRECIST DCR category of responders (CR/PR/SD) and non-responders with SD. **(D)** Predictive performance of PD-L1 and CD274 mRNA expression determined from ROC curves in terms of DCR category. CR, complete response; GC, gastric cancer; iRECIST, immune Response Evaluation Criteria in Solid Tumors; ORR, objective response rate; PD, progressive disease; PD-L1, programmed cell death ligand 1; PR, partial response; ROC, receiver operating characteristics; qRT-PCR, quantitative reverse transcription-polymerase chain reaction; SD, stable disease; UC, urothelial carcinoma.

Anti-PD-1/PD-L1 responders (CR/PR, n = 16) and non-responders (PD/SD, n = 33) were identified using the iRECIST category of ORR. The expression of PD-L1 (P = 0.010) and high *CD274* mRNA expression (P < 0.001) were significantly associated with the response to immunotherapy. The ROC curve for the predictive performance of PD-L1 IHC and mRNA expression of *CD274* at exon 1–2 was discriminatory. The AUC and 95% confidence intervals (CIs) were 0.76 (0.61–0.91) for PD-L1 and 0.75 (0.59–0.91) for mRNA expression of *CD274* exon 1–2. These findings were similar using the iRECIST category of DCR (CR/PR/SD, n = 30 and PD, n = 19). Furthermore, PD-L1 expression (P = 0.015) and high *CD274* mRNA expression (P = 0.038) predicted responses to immunotherapy with AUCs of 0.70 (0.55–0.86) and 0.68 (0.53–0.83), respectively. In GC, the expression of PD-L1 (P = 0.002) and high CD274 mRNA expression (P=0.041) were significantly associated with the response to immunotherapy. In UC, the expression of PD-L1 (P = 0.147) and high CD274 mRNA expression (P=0.008) did not reach statistical significance in predicting response to immunotherapy (**Figure 3A**). The ROC curve for the predictive performance of PD-L1 IHC and mRNA expression of CD274 at exon 1–2 was discriminatory. In GC, the AUC and 95% confidence intervals (CIs) were 0.80 (0.63–0.97) for PD-L1 and 0.69 (0.47–0.92) for mRNA expression of CD274 exon 1–2. In UC, the AUC and 95% Cis were 0.68 (0.36–0.99) for PD-L1 and 0.87 (0.67–1.00) for mRNA expression of CD274 exon 1–2 (**Figure 3B**). These findings were similar using the iRECIST category of DCR (CR/PR/SD, n = 15 and PD, n = 18) in GC. PD-L1 expression (P = 0.008) and high CD274 mRNA expression (P = 0.017) predicted responses to immunotherapy with AUCs of 0.73 (0.56–0.90) and 0.71 (0.53–0.90), respectively, in GC. In UC, anti-PD-1/PD-L1 responders (n = 15) and non-responders (n = 1) were identified using the iRECIST category of DCR. PD-L1 expression (P = 0.375) and high CD274 mRNA expression (P = 0.250) predicted responses to immunotherapy with AUCs of 0.67 (0.43–0.91) and 0.90 (0.71–1.00), respectively (**Figures 3C, D**).

### Correlations Between Survival and PD-L1 Immunohistochemical and qRT-PCR Results

**Figure 4:**
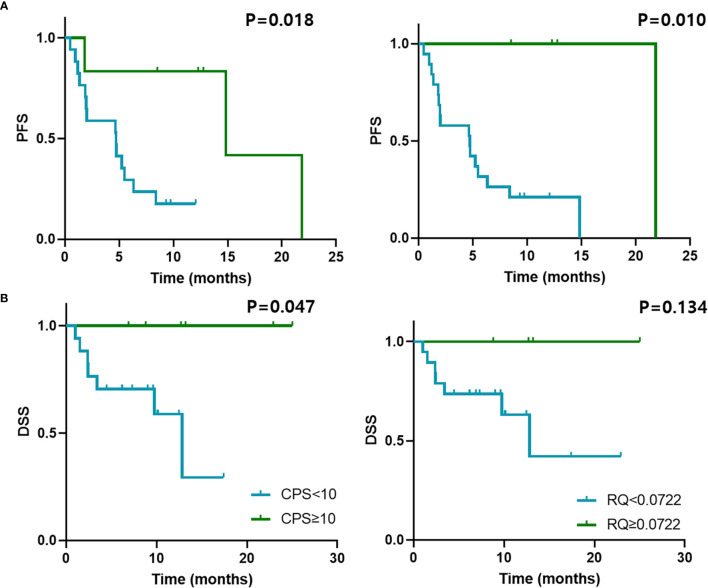
Survival outcomes and qRT-PCR results of GC treated with anti-PD-1/PD-L1. Kaplan–Meier curves of **(A)** PFS and **(B)** DSS of patients with GC treated with anti-PD-1/PD-L1 according to PD-L1 CPS cut-off 10 and *CD274* mRNA expression determined by qRT-PCR with cut-off 0.0722. PFS, progression-free survival; DSS, disease-specific survival.

The PFS was closely associated with PD-L1 expression (P = 0.018) and high CD274mRNA expression spanning the exon 1–2 junction (P = 0.010) in GC (**Figure 4A**). The association was also similar between DSS and PD-L1 expression (P = 0.047). However, DSS was not significantly associated with mRNA expression (P = 0.134); **Figure 4B**). The expression of PD-L1 was significantly associated with PFS (P = 0.016) and DSS (P = 0.009) in UC, whereas the CD274mRNA expression at exon 1–2 junction did not significantly correlate with PFS and DSS (**Supplementary Figure S3**).

### Clinical Value of PD-L1 IHC and qRT-PCR 

The clinical value of PD-L1 assessment with IHC and qRT-PCR was compared using the standard parameters of sensitivity, specificity, PPV, NPV, and accuracy (**Table 2**). We used two cut-offs for GC samples (CPS ≥ 1% and 10%; RQ ≥ 0.0276 and ≥ 0.0772) to ensure the optimal performance to predict responses for immunotherapy. The CPS ≥ 1% for PD-L1 was the most sensitive (90%), and qRT-PCR with a RQ cutoff of 0.0722 was the most specific (100%) in GC. The sensitivity was highest in GC samples with CPS ≥ 1 (90%) although the PPV was very low (50%). The sensitivity (66.7%) and specificity (90%) of detecting UC were higher with qRT-PCR and the AUC values higher than those in PD-L1 IHC.

**Table 2:** Comparison of clinical applicability between IHC PD-L1 and qRT-PCR results.

Prediction Method	Sensitivity	Specificity	PPV	NPV	AUC (95% CI)
Gastric cancer IHC ≥ 1%	90.0%	60.9%	50.0%	93.3%	0.75 (0.58-0.93)
Gastric cancer RQ ≥ 0.0276	60.0%	60.9%	40.0%	77.8%	0.60 (0.39-0.82)
Gastric cancer IHC ≥ 10%	50.0%	87.0%	62.5%	80.0%	0.69 (0.47-0.90)
Gastric cancer RQ ≥ 0.0772	50.0%	100.0%	100.0%	82.1%	0.75 (0.54-0.96)
Urothelial carcinoma IHC ≥ 5%	50.0%	70.0%	50.0%	70.0%	0.60 (0.30-0.90)
Urothelial carcinoma RQ ≥ 0.0480	66.7%	90.0%	80.0%	81.8%	0.78 (0.52-1.00)

AUC, area under ROC curve; IHC, immunohistochemistry; ROC, receiver operating characteristics; RQ, relative quantification; qRT-PCR, quantitative real-time polymerase chain reaction.

## Discussion

The expression of PD-L1 is one of the most studied biomarkers to predict the responses to ICI and one of the most controversial biomarkers to be introduced into clinical practice (3). Despite evidence showing that technological and histological variability limit clinical its utility (2, 22), four IHC-based tests have been approved for guiding treatment decisions regarding patients with multiple tumor types. The wide range of FDA-approved assays with differential sensitivity and scoring systems (23) and the lack of harmonization among them (24) have led to confusion in pathology laboratories (25). In GC, pembrolizumab exhibited favorable efficacy in PD-L1-positive patients (KEYNOTE-059) (26). Owing to the results, pembrolizumab was approved for PD-L1-positive GC patients in second- or later-line treatment by the FDA. However, the predictive value of PD-L1 expression in GC was challenged by other clinical trials (27–29). In UC, five PD-1/PD-L1 inhibitors are approved for treatment of locally advanced or metastatic UC. Due to restrictions by the FDA, first-line treatment with Atezolizumab and Pembrolizumab in platinum-ineligible patients requires PD-L1 IHC. In the second-line setting, all drugs are approved without PD-L1 IHC testing (30). PD-L1 IHC tests used in clinical trials of UC immunotherapy include the 28-8 pharmDx (Nivolumab), the 22C3 pharmDx (Pembrolizumab), Ventana SP142 (Atezolizumab), and the Ventana PD-L1 SP263 assays (Durvalumab). Here, we measured PD-L1 mRNA expression using qRT-PCR and compared the results with FDA-approved PD-L1 IHC assays for GC and UCs. We found that CD274 mRNA expression spanning exon 1–2 closely correlated with PD-L1 IHC and predicted responses to ICIs.

Although PD-L1 IHC measured by IHC is a predictive biomarker of responses to ICIs (22), whether an alternative methodology could validate PD-L1 utility as a predictive biomarker has remained unclear (3). Much effort has been directed towards evaluating whether RNA-based PD-L1 assays could replace PD-L1 IHC as a biomarker to predict responses to ICI (**Table 3**) (3, 31–33, 35–38). Unlike IHC, qRT-PCR or RNA sequencing quantifies the number of mRNA transcripts expressed in an entire tumor without subjective scoring methods or cell type discrimination (3). Recently, various omics-based approaches have been undertaken to identify both tumor intrinsic and extrinsic factors which can serve as predictive biomarkers to ICB (39). Wu et al. reported that high-throughput gene expression data would further help prioritize important biomarkers and potential therapeutic targets for combination treatments with anti-PD-1 therapy for a given cancer type (39). Chen et al. also found that gene expression profiles between responder and non-responder are not significantly different for pre-treatment samples, but much more significantly for on-treatment samples (40). Our results also confirmed that *CD274* mRNA expression measured by qRT-PCR closely correlated with PD-L1 IHC measured using FDA-approved assays. Kowanetz et al. also showed that *CD274* mRNA expression had predictive value for responses to atezolizumab in UC (41). Although our patient cohort was small, we found that high *CD274* mRNA expression determined by qRT-PCR predicted the responses of all 49 patients to immunotherapy with an AUC of 0.75, which was similar to that of PD-L1 IHC (0.76). Objective qRT-PCR assays are operator independent, and can resolve major disadvantages associated with PD-L1 IHC such as assay variance between vendors, subjective assessment by pathologists, and operator-dependent variations in results (42). Therefore, evaluating *CD274* mRNA expression by qRT-PCR has potential as a diagnostic test with easy standardization and a rapid turnaround time.

**Table 3:** Comparison of published CD274 mRNA expression and PD-L1 IHC data.

Study No.	Authors	Years	Cancer Type	Method	Immune checkpoint inhibitor	AUC response to ICI	Cutoff	Patients(n)
	Present study	2021	GC and UC	qRT-PCR and IHC (22C3 and SP142)	Nivolumab, pembrolizumab, and atezolizumab	Overall AUC 0.75 ORR vs. 0.76 by IHC	CPS cutoff 1, 10%, and IC 5%	49
1	Tsimafeyeu et al. (31)	2020	NSCLC	qRT-PCR and IHC (22C3, SP142, SP263)	NA	NA	IHC TC cutoff 10%	437
2	Xiao et al. (32)	2019	ccRCC	qRT-PCR and IHC (E1L3N)	NA	PFS and OS only	IHC TC cutoff 5%	242
3	Conroy et al. (3)	2019	Melanoma, RCC and NSCLC	RNAseq and IHC (22C3, 28-8)	Nivolumab, pembrolizumab, and atezolizumab	Overall 73% ORR vs. 56% by IHC	TPS cutoff 1, 50%, and CPS 1%	209
4	Duncan et al. (33)	2019	NSCLC, HNSCC, and UC	IHC (SP263) and RNAscope	NA	NA	IHC TC 25%	86
5	Vannitamby et al. (34)	2019	NSCLC	qRT-PCR, ddPCR and IHC	NA	NA	IHC TC cutoff 1%	28
6	Tretiakova et al (35)	2018	Bladder carcinoma	RNAscope and IHC (22C3, 28-8, E1L3N, and SP142)	NA	NA	NA	156
7	Erber et al. (36)	2017	NSCLC	qRT-PCR and IHC (E1L3N and 28-8)	NA	NA	IHC TC cutoff 50%	22
8	Shent et al. (37)	2014	Osteosarcoma	qRT-PCR and IHC (B7-H1)	NA	NA	NA	38

AUC, area under the receiver operator characteristics curve; ccRCC, clear cell renal cell carcinoma; GC, gastric cancer; HNS CC, head and neck squamous cell carcinoma; ICI, immune checkpoint inhibitor; IHC, immunohistochemistry; NA, not applicable; NSCLC, Non-small cell lung cancer; qRT-PCR, quantitative real-time polymerase chain reaction; SCC, squamous cell carcinoma; TC, tumor cell; UC, urothelial carcinoma.

One limitation of this study is that it is a single-institutional retrospective investigation of a relatively small sample of patients treated with immunotherapy. We plan to validate our results in a prospective study. Another limitation is that we analyzed patients with GC and UC treated with various individual and combined immunotherapeutic agents in the same cohort. Although gastric and urothelial carcinomas are quite different in their nature, however, in predicting responses for immunotherapy using PD-L1 IHC, CPS is used in interpretation and both cancers were approved relatively early for immunotherapy. Therefore, we decided to study both gastric and urothelial carcinomas. Future studies could address this issue by evaluating patients with GC and UC who receive uniform treatment.

In conclusion, *CD274* mRNA expression measured by qRT-PCR closely correlated with PD-L1 IHC measured using FDA-approved assays and predicted the responses of patients with GC or UC to ICBs.

## Data Availability Statement

The datasets presented in this study can be found in online repositories. The names of the repository/repositories and accession number(s) can be found in the article/**Supplementary Material**.

## Ethics Statement

The studies involving human participants were reviewed and approved by the Institutional Review Board guidelines of the Samsung Medical Center (IRB 2018-09-041-001). The patients/participants provided their written informed consent to participate in this study.

## Author Contributions

SK and K-MK designed and supervised the study. SK, YH, GK and K-MK collected tissue samples and clinical data and performed histopathological examination. SK, YH, and K-MK analyzed the data. SK, YH, GK and K-MK conducted the experiments. SK and K-MK wrote the draft. SK, YH, GK and K-MK revised the manuscript. All authors reviewed and approved the final version of the manuscript.

## Funding

This work was supported by the Basic Science Research Program through the National Research Foundation of Korea (NRF), funded by the Ministry of Science and ICT (NRF-2017R1A2B4012436), and a grant from the Korea Health Technology R&D Project through the Korea Health Industry Development Institute (KHIDI), funded by the Ministry of Health & Welfare, Republic of Korea (grant numbers: HR20C0025 and HI21C1137).

## Conflict of Interest

The authors declare that the research was conducted in the absence of any commercial or financial relationships that could be construed as a potential conflict of interest.

## Publisher’s Note

All claims expressed in this article are solely those of the authors and do not necessarily represent those of their affiliated organizations, or those of the publisher, the editors and the reviewers. Any product that may be evaluated in this article, or claim that may be made by its manufacturer, is not guaranteed or endorsed by the publisher.
