# Supplementary material for: Expression of CD274 mRNA Measured by qRT-PCR Correlates With PD-L1 Immunohistochemistry in Gastric and Urothelial Carcinoma
Source: Front Oncol. 2022 Apr 27;12:856444. doi: 10.3389/fonc.2022.856444 (PMC9094617; doi:10.3389/fonc.2022.856444)
Supplement: Supplementary file 1 [file DataSheet_1.pdf]

Supplementary Table S1: Samples (n=49) sorted within tumor type by PD-L1 test

Patient No.	Tumor Type	Immune checkpoint inhibitor	TNM stage	Stage	Objective Response	CPS	IC	PD-L1 IHC antibody clone	CD274 RQ exon 1-2, 0.0722 cut-off	CD274 RQ exon1-2, 0.0480 cut-off	PD-L1 IHC CPS 1% cut-off	PD-L1 IHC CPS 10% cut-off	PD-L1 IHC IC 5% cut-off
1	GC	Pembrolizumab	M1	IV	CR	0		22C3	<0.0722		<1%	<10%	
2	GC	Pembrolizumab	pT4b N3a	IIIa	CR	80		22C3	≥0.0722		≥1%	≥10%	
3	GC	Pembrolizumab	M1	IV	PR	2		22C3	<0.0722		≥1%	<10%	
4	GC	Nivolumab	pT4a N3a	IIIb	PR	2		22C3	<0.0722		≥1%	<10%	
5	GC	Nivolumab	M1	IV	PR	5		22C3	<0.0722		≥1%	<10%	
6	GC	Pembrolizumab	M1	IV	PR	1		22C3	<0.0722		≥1%	<10%	
7	GC	Pembrolizumab	M1	IV	PR	11		22C3	≥0.0722		≥1%	≥10%	
8	GC	Pembrolizumab	pT3 N1	IIb	PR	80		22C3	≥0.0722		≥1%	≥10%	
9	GC	Pembrolizumab	pT4b N3a	IIIa	PR	90		22C3	≥0.0722		≥1%	≥10%	
10	GC	Pembrolizumab	pT4a N3b	IIIb	PR	95		22C3	≥0.0722		≥1%	≥10%	
11	GC	Pembrolizumab	pT4a N0	IIb	SD	3		22C3	<0.0722		≥1%	<10%	
12	GC	Pembrolizumab	M1	IV	SD	5		22C3	<0.0722		≥1%	<10%	
13	GC	Durvalumab	pT4a N2	IIIa	SD	1		22C3	<0.0722		≥1%	<10%	
14	GC	Pembrolizumab	M1	IV	SD	0		22C3	<0.0722		<1%	<10%	
15	GC	pembrolizumab	M1	IV	SD	0		22C3	<0.0722		<1%	<10%	
16	GC	Nivolumab	pT4a N3b	IIIb	PD	0		22C3	<0.0722		<1%	<10%	
17	GC	Durvalumab	M1	IV	PD	3		22C3	<0.0722		≥1%	<10%	
18	GC	Pembrolizumab	M1	IV	PD	0		22C3	<0.0722		<1%	<10%	
19	GC	Durvalumab	M1	IV	PD	2		22C3	<0.0722		≥1%	<10%	
20	GC	Nivolumab	M1	IV	PD	12		22C3	<0.0722		≥1%	≥10%	
21	GC	Nivolumab	pT4a N3a	IIIb	PD	0		22C3	<0.0722		<1%	<10%	
22	GC	Nivolumab	M1	IV	PD	0		22C3	<0.0722		<1%	≥10%	
23	GC	Nivolumab	pT3 N1	IIb	PD	0		22C3	<0.0722		<1%	<10%	
24	GC	Durvalumab	pT4a N3a	IIIb	PD	0		22C3	<0.0722		<1%	<10%	
25	GC	Nivolumab	M1	IV	PD	0		22C3	<0.0722		<1%	≥10%	
26	GC	Nivolumab	pT4b N2	IIIb	PD	0		22C3	<0.0722		<1%	<10%	
27	GC	Pembrolizumab	M1	IV	PD	0		22C3	<0.0722		<1%	<10%	
28	GC	Nivolumab	rpT2 N1	IV	PD	0		22C3	<0.0722		<1%	<10%	
29	GC	Nivolumab	pT4a N3a	IIIb	PD	12		22C3	<0.0722		≥1%	≥10%	
30	GC	Pembrolizumab	M1	IV	PD	25		22C3	<0.0722		≥1%	≥10%	
31	GC	pembrolizumab	pT3 N1	IIb	PD	0		22C3	<0.0722		<1%	<10%	
32	GC	Nivolumab	pT3 N3a	IIIb	PD	0		22C3	<0.0722		<1%	<10%	
33	GC	Nivolumab	M1	IV	PD	3		22C3	<0.0722		≥1%	<10%	
34	Urothelial	Nivolumab	T2	II	CR		0	SP142	0.01153	<0.0480			<5%

35	Urothelial	Atezolizumab	pT3b N2	IIIb	CR		50	SP142	0.17342	≥0.0480			≥5%
36	Urothelial	Atezolizumab	pT3b N2	IIIb	CR		50	SP142	0.51228	≥0.0480			≥5%
37	Urothelial	Nivolumab	T2	II	PR		1	SP142	0.05241	≥0.0480			<5%
38	Urothelial	Nivolumab	T2	II	PR		2	SP142	0.02822	<0.0480			<5%
39	Urothelial	Atezolizumab	pT3 N1	IIIa	PR		95	SP142	0.83284	≥0.0480			≥5%
40	Urothelial	Atezolizumab	pT2 N0	II	SD		0	SP142	0.00000	<0.0480			<5%
41	Urothelial	Atezolizumab	pT2 Nx	II	SD		0	SP142	0.01535	<0.0480			<5%
42	Urothelial	Atezolizumab	pT2a	II	SD		1	SP142	0.02038	<0.0480			<5%
43	Urothelial	Atezolizumab	ypT3 N2	IIIb	SD		1	SP142	0.04487	<0.0480			<5%
44	Urothelial	Atezolizumab	pT2	II	SD		1	SP142	0.05112	≥0.0480			<5%
45	Urothelial	Atezolizumab	pT3 Nx	IIIa	SD		2	SP142	0.00000	<0.0480			<5%
46	Urothelial	Atezolizumab	pT3 Nx	IIIa	SD		2	SP142	0.01245	<0.0480			<5%
47	Urothelial	Atezolizumab	pT2	II	SD		15	SP142	0.00000	<0.0480			≥5%
48	Urothelial	Atezolizumab	pT2	II	SD		15	SP142	0.01742	<0.0480			≥5%
49	Urothelial	Atezolizumab	pT3a N2	IIIb	PD		5	SP142	0.00000	<0.0480			<5%

RQ, relative quantification; IHC, immunohistochemistry; GC, gastric cancer; CR, complete response; PR, partial response; SD, stable disease; PD, progressive disease; CPS, combined positive score; IC, immune cell

Supplementary Table S2. PD-L1 IHC and qRT-PCR results of 100 samples.

Results	Test	GC	UC
Immunohistochemistry		22C3 pharmDx	SP142
≥1 CPS	IHC	32 (54.2%)	
<1 CPS	IHC	27 (45.8%)	
≥10 CPS	IHC	13 (22.0%)	
<10 CPS	IHC	46 (78.0%)	
≥5 IC	IHC		12 (29.3%)
<5 IC	IHC		29 (70.7%)
Cut-offs for qRT-PCR			
≥0.0276 RQ exon 1–2	CPS ≥1	22 (37.3%)	
<0.0276 RQ exon 1–2		37 (62.7%)	
≥0.0563 RQ exon 3–4		34 (57.6%)	
<0.0563 RQ exon 3–4		25 (42.4%)	
0.0511≥ RQ exon 5–6		14 (23.7%)	
<0.0511 RQ exon 5–6		45 (76.3%)	
≥0.0722 RQ exon 1–2	CPS ≥ 10	13 (22.0%)	
<0.0722 RQ exon 1–2		46 (78.0%)	
≥0.1966 RQ exon 3–4		9 (15.3%)	
<0.1966 RQ exon 3–4		50 (84.7%)	
≥0.0528 RQ exon 5–6		13 (22.0%)	
<0.0528 RQ exon 5–6		46 (78.0%)	
≥0.048 RQ exon 1–2	IC ≥ 5		12 (29.3%)
<0.048 RQ exon 1–2			29 (70.7%)
≥0.1966 RQ exon 3–4			7 (17.1%)
<0.1966 RQ exon 3–4			34 (82.9%)
≥0.0528 RQ exon 5–6			13 (31.7%)
<0.0528 RQ exon 5–6			28 (68.3%)
	**Total**	**59**	**41**

CPS, combined positive score; GC, gastric cancer; IHC, immunohistochemistry; qRT-PCR, quantitative real-time polymerase chain reaction; UC, urothelial carcinoma.

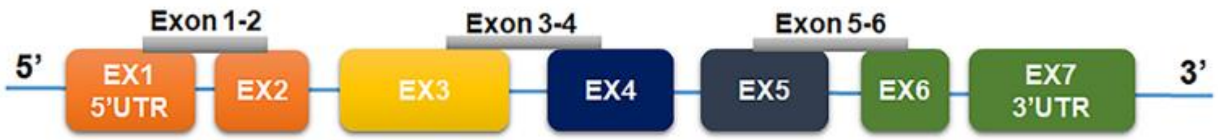

Supplementary Figure S1. Schematic view of *CD274* and TaqMan probes spanning exon 1–2, 3–4, and 5–6 boundaries.

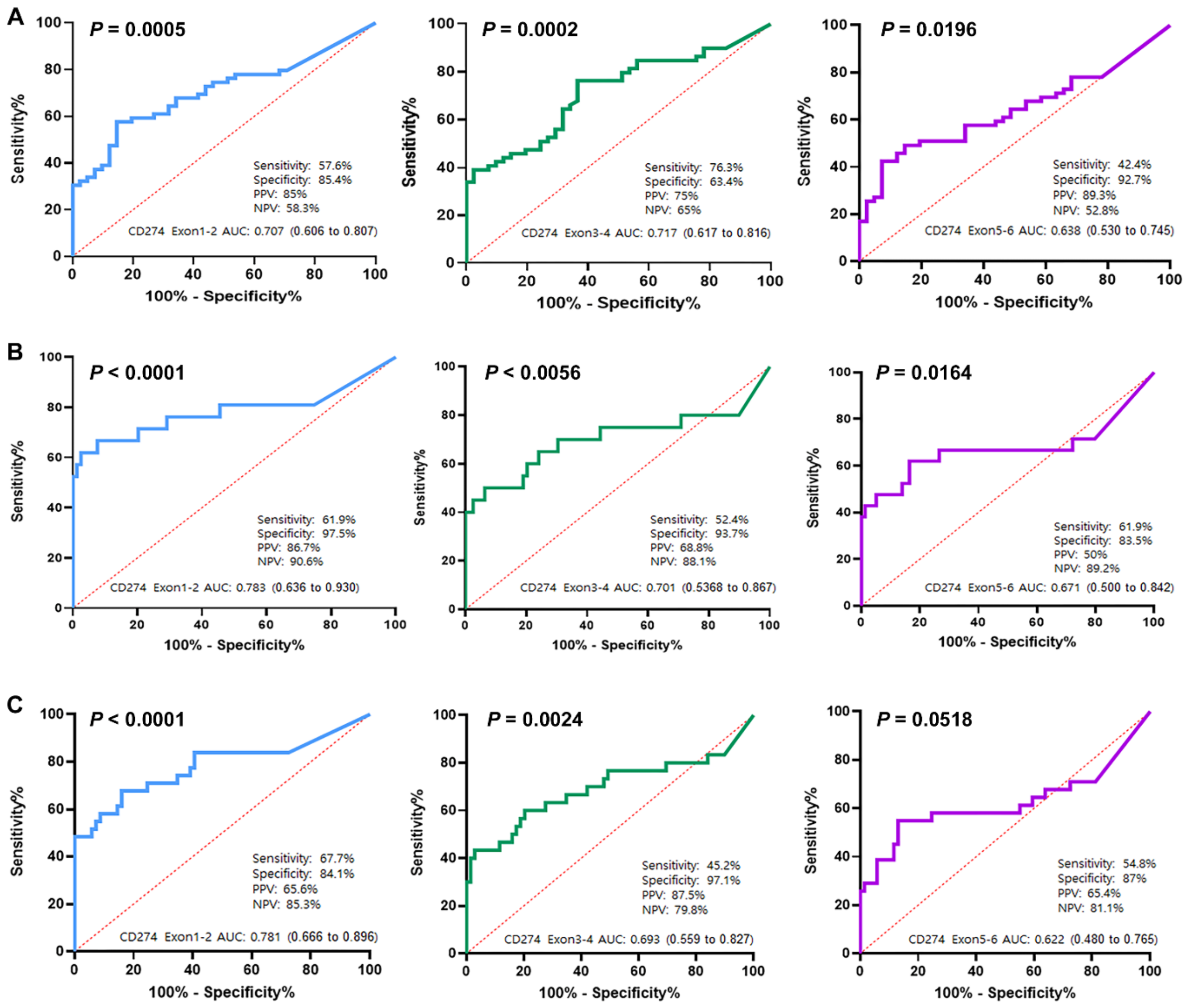

Supplementary Figure S2. Predictive performance of CD274 exons 1–2, 3–4, and 5–6.

Predictive performance of CD274 exons using ROC curves in terms of PD-L1 CPS cut-offs of (A) 1 and (B) 10 for GC and (C) PD-L1 IC cut-offs of 5 for UC. CPS, combined positive score GC, gastric cancer; IC, immune cell; PD-L1, programmed death-ligand 1; ROC, receiver operating characteristics; UC, urothelial carcinoma.

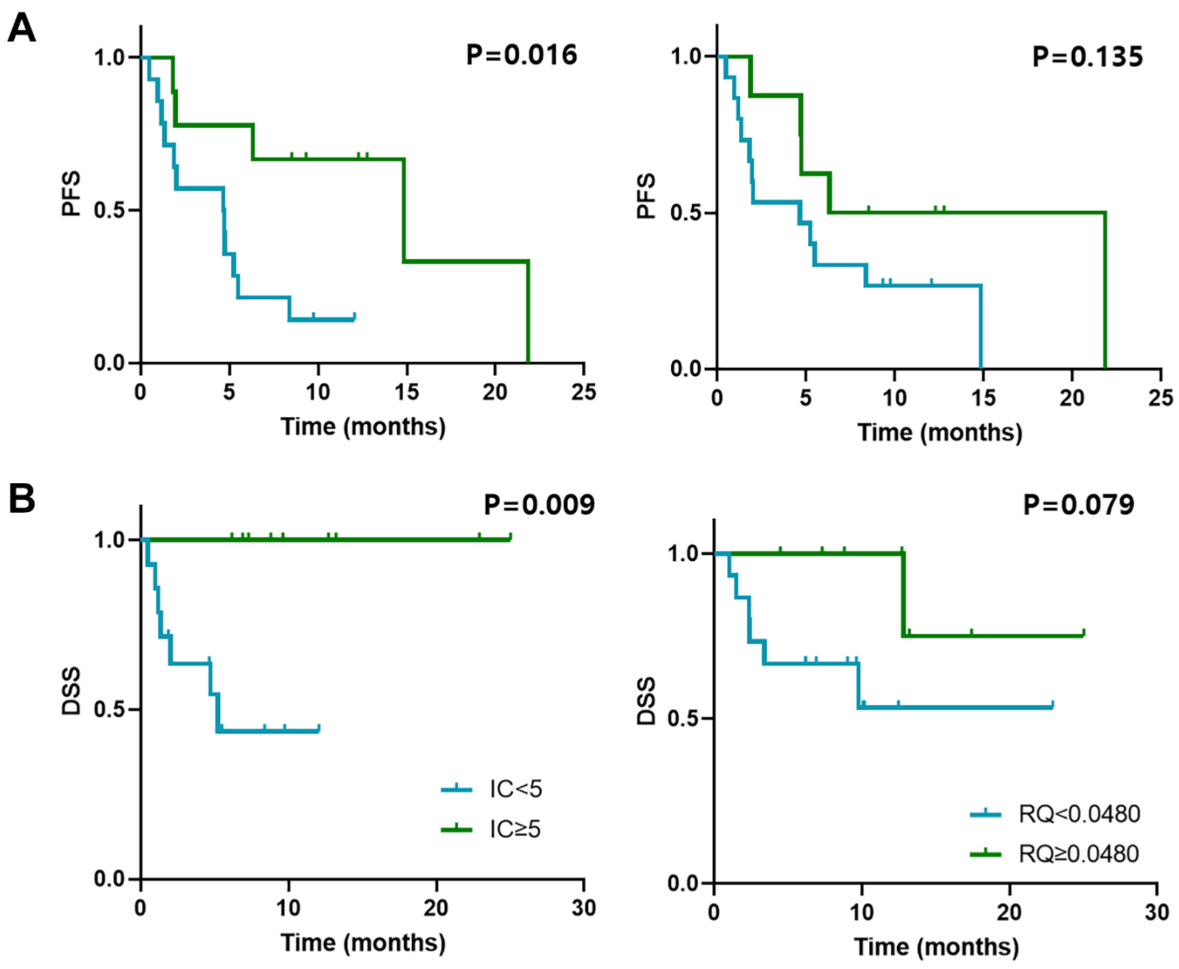

Supplementary Figure S3. Results of qRT-PCR and survival outcomes of UC treated by PD-1/PD-L1. Kaplan–Meier analysis of (A) PFS and (B) OS of patients with UC treated by anti-PD-1/PD-L1 according to PD-L1 IC cut-off at 5 and *CD274* mRNA expression by qRT-PCR cut-off at 0.0480. IC, immune cell; DSS, disease-specific survival; PD-L1, programmed cell death ligand 1; PFS, progression-free survival; qRT-PCR, quantitative reverse transcription-polymerase chain reaction; UC, urothelial carcinoma.
